# Navigating Multiple Challenges: Malnutrition and Nephrotoxic Drug Effects in a Non-verbal Child With Autism Spectrum Disorder Requiring Dialysis

**DOI:** 10.7759/cureus.56951

**Published:** 2024-03-26

**Authors:** Yusuke Matsuura, Jan Fune, Lena Ngai

**Affiliations:** 1 Pediatrics, Mount Sinai Hospital, New York, USA; 2 Pediatrics, Icahn School of Medicine at Mount Sinai, New York, USA; 3 Pediatrics/Hospital Medicine, Mount Sinai Hospital, New York, USA; 4 Pediatrics/Hospital Medicine, Icahn School of Medicine at Mount Sinai, New York, USA; 5 Pharmacy, Mount Sinai Hospital, New York, USA

**Keywords:** acute kidney injury, nephrotoxic medications, nephrotoxic drugs, pediatrics, vancomycin, nsaids, hypoalbuminemia, autism spectrum disorder, asd, aki

## Abstract

Acute kidney injury (AKI) is a common medication adverse event, particularly in patients with pre-existing medical conditions taking nephrotoxic medications. However, little is known about the differences in the risk of nephrotoxic medication-related complications in children with autism spectrum disorder (ASD) compared to the general pediatric population.

A nine-year-old non-verbal boy with ASD was hospitalized for scrotal cellulitis requiring vancomycin and piperacillin/tazobactam due to a lack of clinical response to cephalosporins. His history is significant for being an extremely selective eater, and his appetite decreased over four months prior to presentation. Poorly controlled scrotal pain, despite acetaminophen use, was suspected based on his facial expressions and maternal assessment, especially considering his non-verbal status. Consequently, a non-steroidal anti-inflammatory drug was initiated. The hospital course was complicated by the development of a scrotal abscess, minimal enteral intake, hypoalbuminemia-induced intravascular dehydration, oliguria, and generalized edema. His creatinine increased to 5.11 mg/dL from 0.51 mg/dL despite early discontinuation of nephrotoxic medications and fluid resuscitation, which led to hemodialysis due to worsening AKI. Subsequently, urinary output and edema improved. Creatinine improved to <1 mg/dL with careful creatinine monitoring and concomitant furosemide and albumin infusion in the pediatric intensive care unit.

Children with comorbidities, such as malnutrition, who require nephrotoxic medications, need extra attention. Implementing clinical decision support tools or quality improvement programs can promote the prevention of nephrotoxic medication exposure and decrease the incidence of AKI. An alert within an electronic health record system for multiple nephrotoxic drugs and daily multidisciplinary huddles during patient-centered rounds could help reduce and eliminate adverse events. In particular, for non-verbal patients or those with limited communication skills, such as children with ASD, rigorous and close monitoring of vital signs, physical condition, pain, medication intake, and lab results, in addition to a nephrotoxic medication screening and notification system, should be key to optimizing patient care.

## Introduction

Acute kidney injury (AKI) is a well-known common adverse event associated with commonly used nephrotoxic medications such as vancomycin, acyclovir, gentamicin, and non-steroidal anti-inflammatory drugs (NSAIDs), as well as lesser-known drug-induced AKI medications such as acetaminophen [1,2]. The risk of this adverse drug reaction is even more likely when using a combination of nephrotoxic drugs with patients who have comorbidities. Over 70% of patients with autism spectrum disorder (ASD) have at least one comorbid disease, and 41% have two or more [3]. 46-89% of children with ASD have some feeding difficulties [4], which can lead to malnutrition and hypoalbuminemia in severe cases. Hypoalbuminemia causes loss of oncotic pressure and third spacing, which could lead to renal hypoperfusion. There is minimal data regarding the risk of developing nephrotoxic medication sequelae and management challenges in patients with ASD who have pre-existing medical conditions. Here, we report a complicated pediatric case that resulted in a patient with ASD and hypoalbuminemia admitted for scrotal cellulitis who required dialysis to treat AKI secondary to exposure to nephrotoxic medications.

## Case presentation

**Figure 1:**
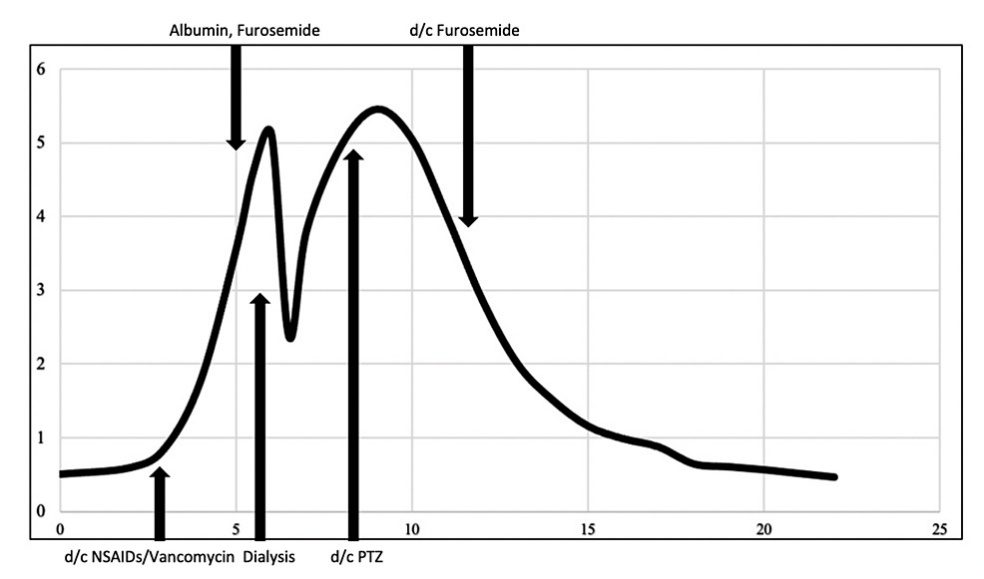
Trend of serum creatinine and major events X-axis: Day of hospitalization, Y-axis: creatinine (mg/dL), d/c: discontinued, PTZ: piperacillin/tazobactam; NSAIDs: non-steroidal anti-inflammatory drugs

A nine-year-old male with hypothyroidism, ASD associated with selective eating habits and non-verbal status, and two recent urinary tract infections (UTIs) four months and a month prior presented to the outpatient clinic with four days of fever and scrotal edema and discomfort. Cefdinir (7 mg/kg every 12 hours) was subsequently prescribed for a presumed recurrent UTI based on the result of urinalysis. The patient was brought to the emergency department due to persistent fever and worsening scrotal discomfort despite having taken cefdinir for two days. A significant loss of appetite and a decrease in urinary frequency to once or twice a day were appreciated. Upon admission, his weight was 38.6 kg, he was afebrile, and other vital signs were normal for his age, except for tachycardia (120 beats/minute); his pre-illness weight was 45.0 kg. Physical findings were notable for perineal firmness and induration with erythematous scrotum. At his baseline, he was unable to follow simple verbal commands and could only produce vowel sounds without any purposeful meaning. Blood work revealed a high WBC count of 17,100 /uL (normal range 3,700-10,500 /uL), elevated CRP of 12.1 mg/L (normal range <10 mg/L), BUN of 6 mg/dL (normal range 6-23 mg/dL), creatinine of 0.51 mg/dL (normal range <0.7 mg/dL), (Figure 1), and serum potassium of 3.6 mEq/L (normal range 3.5-5.2 mEq/L). The urinalysis revealed numerous white blood cells, 4-10 red blood cells/HPF, urine protein < 30 mg/dL, no bacteria, negative for nitrite, and positive for leukocyte esterase. Ultrasound ruled out testicular torsion, epididymitis, and orchitis but demonstrated an edematous scrotum.

A cephalosporin-resistant pathogen causing scrotal cellulitis and UTIs were suspected given his presentation and lack of response to cefdinir. Intravenous (IV) vancomycin (15 mg/kg every eight hours) and piperacillin/tazobactam (PTZ) (100 mg/kg every six hours) were started for empiric coverage. Further history revealed that he was an extremely selective eater at baseline; diet mainly consisted of only white bread, chicken, rice, apple juice, and cola, with no intake of other foods. His dietary intake had worsened more over the four months since his first UTI diagnosis. Due to a significant weight loss of 14.2% in four months, malnutrition was suspected. Subsequently, he was started on oral potassium chloride 40 mEq and maintenance IV fluid containing dextrose and sodium chloride 0.9 %. The pain from the perineal and scrotal lesions was considered to be poorly controlled despite acetaminophen use, as indicated by his grimacing and observations made by his mother; consequently, oral ibuprofen was added to his pain management regimen. The vancomycin drug level was within the acceptable target trough range (9.0 and 12.3 mcg/mL on day 1 and day 2; target range 8-12 mcg/mL).

Blood work on day 3 of hospitalization revealed an elevated vancomycin trough level of 19.0 mcg/mL and an elevated creatinine of 0.88 mg/dL (1.72 times above his first workup of 0.52 mg/dL), indicating the development of AKI. The urine culture collected upon admission showed no growth after 48 hours. Ibuprofen was immediately discontinued, and vancomycin was switched to IV linezolid (10 mg/kg every 12 hours). Despite these changes, he developed facial edema with persistent oliguria on day 4 with a creatinine of 1.79 mg/dL (3.5 times above baseline). To evaluate the adverse drug reaction, the Naranjo Adverse Drug Reaction Probability Scale was used, resulting in a score of 5. This score indicates a “probable” reaction (a score of 9 or more is “definite,” 5-8 is “probable,” 1-4 is “possible,” and 0 or less is “doubtful”). WBC was still up-trending to 29,200 /uL, with an elevated CRP of 39.1 mg/L. Down-trending serum potassium of 2.6 mEq/L and low albumin of 2.4 g/dL (normal range >4.1 g/dL) were found. Consequently, albumin was infused, and he was transferred to the intensive care unit (ICU).

A follow-up ultrasound on hospital day 5 showed normal kidney and bladder anatomy but revealed a new 6 x 4 x 4 cm sized abscess in the left scrotum. ICU management included abscess drainage, IV potassium, furosemide, albumin infusion, and hydromorphone for persistent scrotal pain. However, oliguria did not improve, and generalized edema characterized by 3 kg of weight gain since admission was noted. On hospital day 6, creatinine was elevated to 5.11 mg/dL, consistent with AKI stage 3 based on Kidney Disease Improving Global Outcomes (KDIGO) criteria, which required hemodialysis on the same day. Dialysis was stopped after 1 liter of fluid removal due to complications of intradialytic hypotension. IV furosemide was continued after dialysis with close electrolyte monitoring. On hospital day 8, scrotal wound culture grew Enterococcus faecalis, sensitive to ampicillin, and PTZ was discontinued on day 8. Linezolid was switched to oral amoxicillin on day 12, and furosemide was discontinued on day 12 because generalized edema and oliguria improved (urinary output > 2 mL/kg/hour). Creatinine gradually decreased after dialysis and eventually became <1 mg/dL on day 16. All antibiotics were completed on day 19 and the serum potassium level at discharge was 4.5 mEq/L.

Notably, the entire hospital course was complicated by persistent hypoalbuminemia (2.6, 2.7 g/dL on days 13 and 16) and poor oral intake. Assuming that he has synthetic liver dysfunction, ensuring appropriate nutrition is the only real solution for this condition, which necessitated the placement of a nasogastric tube (NG) before discharge. His discharge weight was 37.2 kg, 6 kg lighter than his pre-illness weight, and he was discharged with NG feeding for better nutrition. Five months later, following feeding therapy, his albumin levels increased to 4.8 g/dL, and he regained 3 kg.

## Discussion

The use of three nephrotoxic medications (vancomycin, PTZ, and ibuprofen) in the setting of hypoalbuminemia caused by malnutrition status likely led to complicated AKI in this case. According to AKI KDIGO definition and classification, AKI is defined as any of the following: an increase in serum creatinine by ≥0.3 mg/dL (≥26.5 μmol/L) within 48 hours, an increase in serum creatinine to ≥1.5 times the baseline is also known or presumed to have occurred within seven days, or urine volume <0.5 mL/kg/hour for six hours [5]. After observing a mild elevation of creatinine, kidney function continued to worsen despite the immediate discontinuation of nephrotoxic medications and fluid management, until hemodialysis was performed. Several factors are considered to have contributed to this extreme complication of his clinical course.

Resistance to antibiotics

The scrotal infection did not show a clinical response to initial empiric cephalosporins started for a presumed urinary tract infection, limiting choices for empiric antimicrobial therapy. Dual antibiotic treatments with vancomycin and PTZ were therefore initiated to cover for MRSA, gram-negative, and anaerobic organisms; However, the patient did not respond promptly and developed a scrotal abscess. Definitive antimicrobial treatment was delayed since there was no growth in the urine culture and a pathogen was not identified. Drainage from the scrotal abscess grew Enterococcus faecalis, which explained why the initial antimicrobial treatment was ineffective. The incidence rate of vancomycin-associated AKI ranges from 12.6 % to 27.2 % in pediatric cases. [6]. Although some studies indicate that pro-inflammatory oxidation by vancomycin will cause cell damage [1,2], the mechanism is not fully understood. Most studies define vancomycin-associated AKI as at least two or three consecutive elevations in serum creatinine by 0.5 mg/dL or at least a 50 % increase from baseline, whichever is greater [6]. The increase must be documented after several days of vancomycin therapy, and no alternative explanation for the impairment in glomerular filtration rate. Nephrotoxicity risk factors for vancomycin include total dose, duration, trough level, severity of illness, and concurrent nephrotoxin exposure [6]. Regarding the duration of treatment, most studies have a significant positive association only when vancomycin is used for more than a week [6]. Therefore, the duration was not likely to be an essential factor in this case, given that vancomycin was discontinued within 72 hours. Regarding the trough, a study shows that patients with initial trough values of >10 mcg/mL had a significantly higher incidence of nephrotoxicity than patients with initial trough values of <10 like in this case of 9.0. (22.2% vs. 5.3%; P=.001) [7]. Although the Naranjo Adverse Drug Reaction Probability Scale was a score of 5 in this case, considered a probable adverse drug reaction, estimating the risk of vancomycin-associated AKI was challenging in this case [8]. This was not only because the vancomycin trough and creatinine levels were monitored daily at the same dose and frequency, but also because there were no apparent alarming signs until there was a sudden elevation of the vancomycin trough to 19 mcg/mL on day 3. This is consistent with a study that concluded that monitoring serum vancomycin concentrations is not a valuable predictor of vancomycin-associated AKI in the pediatric population [9].

Finally, concomitant nephrotoxic agents with PTZ likely contributed to his AKI. Although PTZ monotherapy is not considered a nephrotoxic medication in pediatric studies, concurrent use of vancomycin with PTZ increased the risk of AKI [6]. Although the actual mechanism of enhanced toxicity of vancomycin and PTZ combination is not clear, the rate of AKI is 29% when vancomycin and PTZ are used together, compared to an 11% rate when vancomycin is used with cefepime (P < 0.0001) [10]. Commonly used antimicrobials and other nephrotoxic medications, the mechanism of injury, and the type of clinical presentation are listed in Table 1 [1,2,11,12].

**Table 1:** Commonly used nephrotoxic medications, mechanisms of injury, and clinical presentation Table credits: Lena Ngai NSAIDs: Non-steroid anti-inflammatory drugs

	Medication Examples	Mechanism of Injury	Clinical Presentation
Anti-infectives			
Aminoglycosides	Amikacin, gentamicin, tobramycin	Direct proximal/distal tubule cytotoxicity	Fanconi-like syndrome, Acute tubular necrosis, Electrolyte wasting tubulopathy
Antifungals	Amphotericin B, Caspofungin	Direct distal tubule cytotoxicity	Renal distal tubular acidosis, Mild distal tubulopathy
Antivirals	Acyclovir	Crystalline nephropathy direct tubular cytotoxicity	Nephrolithiasis, Acute interstitial nephritis
	Foscarnet, Cidofovir	Direct proximal tubule cytotoxicity	Acute tubular necrosis
Beta-lactams	Penicillin, Piperacillin/tazobactam, Cephalosporins (i.e., cefuroxime)	Direct proximal tubule cytotoxicity	Acute tubular necrosis
		Glomerular injury	Acute glomerulonephritis
Fluoroquinolones	Ciprofloxacin, Moxifloxacin, Levofloxacin	Crystalline nephropathy	Acute interstitial nephritis
		Tubular damage enhanced cellular immunity	Thrombotic microangiopathy
Glycopeptides	Vancomycin	Direct proximal tubule cytotoxicity	Acute tubular necrosis, Acute interstitial nephritis
Sulfonamides	Trimethoprim/Sulfamethoxazole, Sulfadiazine	Impaired creatinine secretion	Acute interstitial nephritis
		Epithelial sodium channel inhibition crystalline nephropathy	Falsely elevated creatinine, Hyperkalemia
Non-anti-infectives			
Angiotensin-converting enzyme (ACE) inhibitors	Captopril, Enalapril, Lisinopril	Altered intraglomerular hemodynamics	Acute interstitial nephritis
Chemotherapeutic Agents	Cisplatin	Proximal tubulopathies	Acute tubular necrosis, Thrombotic microangiopathy
	Ifosfamide	Proximal tubulopathies	Fanconi-like syndrome
	Methotrexate	Crystalline nephropathy	Acute tubular necrosis
Diuretics	Loop (i.e. Furosemide) thiazide	Altered intraglomerular hemodynamics	Acute interstitial nephritis
Immunosuppressants	Cyclosporine, Tacrolimus	Altered intraglomerular hemodynamics	Chronic interstitial nephritis, Thrombotic microangiopathy
NSAIDs	Ibuprofen, Ketorolac	Inhibit production of vasodilatory prostaglandins with afferent arteriolar vasoconstriction	Acute/chronic interstitial nephritis, glomerulonephritis

Selective eating leading to malnutrition and intravascular dehydration

At baseline, he had a very limited diet in the setting of ASD, and his first UTI four months prior to admission exacerbated his decreased appetite. Compared with his pre-illness weight of 43.1 kg, he had lost more than 4 kg at admission. Moreover, hospitalization and hospital meals likely increased his stress level and worsened his oral intake, contributing to progressing hypoalbuminemia. Hypoalbuminemia is defined as <3.5-4.0 g/dL, and his serum albumin level during hospitalization was 2.4 g/dL. While his albumin level was not assessed at the time of admission, it is likely that he had pre-existing hypoalbuminemia. Low albumin level decreases intravascular oncotic pressure, and intravascular fluid shifts to the interstitial space, leading to intravascular volume depletion and edema as in this case. Furthermore, patients with hypoalbuminemia were significantly more likely to develop AKI and progress from AKI to chronic kidney disease (CKD) stage 4 [13]. Adequate hydration before and during vancomycin treatment prevents vancomycin-associated AKI in patients with intravascular dehydration [14]. However, the patient likely did not respond to hydration and continued to suffer from irreversible intravascular dehydration due to hypoalbuminemia.

Pain assessment for the patient in non-verbal status

The accurate assessment of pain was challenging due to the patient's non-verbal baseline status, associated with ASD. For example, his expression of his discomfort was limited to crying, agitation, and facial grimacing. Decision-making of his pain management from these non-verbal expressions primarily depended on his mother’s observation. Initial pain management with acetaminophen failed to control his symptoms and ibuprofen was given as his second pain management option. After the creatinine elevation to 0.88 mg/dL, ibuprofen was immediately discontinued, and the pain was managed with hydromorphone, but the creatinine continued to rise. Although ibuprofen use was associated with a 23% increased risk of pediatric hospital-acquired AKI [15], the rate of NSAIDs associated with AKI in children is small (2.7%) [16]. Symptomatic pediatric patients (not limited to AKI) after ibuprofen intake had a mean ingestion of 440 mg/kg; those who remained asymptomatic had a mean ingestion of 114 mg/kg (P < .001) [17]. In this case, the cumulative ibuprofen dose over 3.5 days was 3,740 mg (97 mg/kg), which did not indicate a high risk. However, pre-existing UTIs and simultaneous medication use might have made him susceptible to developing AKI.

Hypokalemia

Although the concomitant infusion of furosemide and albumin often addresses both hypoalbuminemia-induced intravascular dehydration and intravascular overload due to sudden fluid shifts from the third space, a common adverse effect of furosemide is hypokalemia. Since the patient already had hypokalemia with poor response to supplementation, furosemide was avoided in the earlier phase of his hospitalization. Due to the hesitancy of concomitant albumin and furosemide use, the intravascular fluid shift was not achievable, which might have contributed to his worsening intravascular dehydration and AKI. Since PTZ use is associated with hypokalemia [18], this could serve as a possible explanation for the persistent hypokalemia in this patient, in addition to the factor of malnutrition. Notably, hypokalemia resolved at a similar time when PTZ was discontinued. Furosemide and albumin were co-administered in the ICU on day 5.

From all these above, how can we prevent AKI complications when initiating nephrotoxic medications, especially for children with comorbidities? Since AKI can cause multiorgan failure in some cases, and AKI rate doubles when children receive 3 or more nephrotoxic medications on the same day [19], avoiding the use of three or more nephrotoxic medications or limiting triple medication courses as short as possible are preferable. The Negated by Just-in-Time Action (NINJA) program implemented systematic screening for hospitalized children exposed to high burdens of nephrotoxic medications to assess the development of AKI. Non-critically ill hospitalized children receiving IV aminoglycosides for more than three days, or more than three nephrotoxins simultaneously, were included in the study. Pharmacists recommended daily serum creatinine monitoring for exposed patients after their appearance on the daily screening report (Table 2). As a result, the exposure rate decreased by 38% (from 11.63 to 7.24 exposures per 1000 patient days), and the AKI rate decreased by 64% (from 2.96 to 1.06 episodes per 1000 patient days) [20]. The use of multiple nephrotoxic medications necessitates heightened vigilance and increased laboratory monitoring, especially in children with underlying physical or developmental disabilities and chronic diseases. At present, there is no specific quality improvement project aimed at high-risk hospitalized children like those described in this case. There may be room to implement a more focused approach to assess nephrotoxic medication exposure and AKI risk for children with comorbidities like malnutrition associated with ASD. Formulating more targeted preventive measures could significantly enhance the prevention of nephrotoxic side effects in these high-risk pediatric populations.

**Table 2:** Nephrotoxic medications used in the NINJA program Table credits: Lena Ngai NINJA: Negated by Just-in-Time Action

List of Nephrotoxic Medications		
Anti-infectives		
Acyclovir	Cidofovir	Piperacillin
Ambisome	Colistimethate	Piperacillin/tazobactam
Amikacin	Dapsone	Ticarcillin/clavulanic acid
Amphotericin B	Foscarnet	Tobramycin
Cefotaxime	Ganciclovir	Valacyclovir
Ceftazidime	Gentamicin	Valganciclovir
Cefuroxime	Nafcillin	Vancomycin
Other Medications		
Captopril	Ibuprofen	Lithium
Carboplatin	Ifosfamide	Mesalamine
Cisplatin	Iodixanol	Methotrexate
Cyclosporine	Iohexol	Sirolimus
Enalapril	Iopamidol	Sulfasalazine
Enalaprilat	Ioversol	Tacrolimus
Gadopentetate dimeglumine	Ketorolac	Topiramate
Gadoxetate disodium	Lisinopril	Zonisamide

Regardless, implementing a clinical screening tool in the electronic health record, or manual notification by the pharmacist, or a quality improvement program containing those elements, could be very helpful and impactful. Ideally, a notification system could inform the medical staff of what and how many nephrotoxic medications the patient is receiving to help promote early decisions regarding medication changes and contingency planning to prevent AKI development beforehand. A nephrotoxic medication list readily available during rounds, or a list of patients on nephrotoxic medicines to be discussed during medical team huddles can also be helpful reminders.

## Conclusions

Pediatric patients with pre-existing medical conditions, such as physical and developmental disabilities, or chronic medical conditions like malnutrition-induced hypoalbuminemia, require extra caution in the selection, duration, and combination of nephrotoxic medications. Implementing an alert system within an electronic health record for multiple nephrotoxic drugs, and/or conducting daily multidisciplinary medication huddles during patient-centered rounds, could help prevent adverse events. Additionally, for children with limited communication abilities, such as those with ASD, rigorous and close monitoring of vital signs, physical condition, pain, medication intake, and lab results, along with implementing a screening and notification system for nephrotoxic medications, should be key components in optimizing patient care.

## References

[1] Naughton CA (2008). Drug-induced nephrotoxicity. Am Fam Physician.

[2] Perazella MA, Rosner MH (2022). Drug-induced acute kidney injury. Clin J Am Soc Nephrol.

[3] Simonoff E, Pickles A, Charman T, Chandler S, Loucas T, Baird G (2008). Psychiatric disorders in children with autism spectrum disorders: prevalence, comorbidity, and associated factors in a population-derived sample. J Am Acad Child Adolesc Psychiatry.

[4] Ledford JR, Gast DL (2006). Feeding problems in children with autism spectrum disorders: a review. Focus Autism Other Dev Disabl.

[5] Khwaja A (2012). KDIGO clinical practice guidelines for acute kidney injury. Nephron Clin Pract.

[6] Bamgbola O (2016). Review of vancomycin-induced renal toxicity: an update. Ther Adv Endocrinol Metab.

[7] Lodise TP, Patel N, Lomaestro BM, Rodvold KA, Drusano GL (2009). Relationship between initial vancomycin concentration-time profile and nephrotoxicity among hospitalized patients. Clin Infect Dis.

[8] Naranjo CA, Busto U, Sellers EM (1981). A method for estimating the probability of adverse drug reactions. Clin Pharmacol Ther.

[9] Moffett BS, Morris J, Kam C, Galati M, Dutta A, Akcan-Arikan A (2018). Vancomycin associated acute kidney injury in pediatric patients. PLoS One.

[10] Navalkele B, Pogue JM, Karino S (2017). Risk of acute kidney injury in patients on concomitant vancomycin and piperacillin-tazobactam compared to those on vancomycin and cefepime. Clin Infect Dis.

[11] Nolin TD, Himmelfarb J (2010). Mechanisms of drug-induced nephrotoxicity. Handb Exp Pharmacol.

[12] Pazhayattil GS, Shirali AC (2014). Drug-induced impairment of renal function. Int J Nephrol Renovasc Dis.

[13] Shao M, Wang S, Parameswaran PK (2017). Hypoalbuminemia: a risk factor for acute kidney injury development and progression to chronic kidney disease in critically ill patients. Int Urol Nephrol.

[14] Kan WC, Chen YC, Wu VC, Shiao CC (2022). Vancomycin-associated acute kidney injury: a narrative review from pathophysiology to clinical application. Int J Mol Sci.

[15] Su L, Li Y, Xu R (2021). Association of ibuprofen prescription with acute kidney injury among hospitalized children in China. JAMA Netw Open.

[16] Misurac JM, Knoderer CA, Leiser JD, Nailescu C, Wilson AC, Andreoli SP (2013). Nonsteroidal anti-inflammatory drugs are an important cause of acute kidney injury in children. J Pediatr.

[17] Hall AH, Smolinske SC, Conrad FL, Wruk KM, Kulig KW, Dwelle TL, Rumack BH (1986). Ibuprofen overdose: 126 cases. Ann Emerg Med.

[18] Zaki SA, Lad V (2011). Piperacillin-tazobactam-induced hypokalemia and metabolic alkalosis. Indian J Pharmacol.

[19] Moffett BS, Goldstein SL (2011). Acute kidney injury and increasing nephrotoxic-medication exposure in noncritically-ill children. Clin J Am Soc Nephrol.

[20] Goldstein SL, Mottes T, Simpson K, Barclay C, Muething S, Haslam DB, Kirkendall ES (2016). A sustained quality improvement program reduces nephrotoxic medication-associated acute kidney injury. Kidney Int.

